# Itinéraire thérapeutique, coûts directs, et qualité de vie des patients atteints de gale humaine suivis en dermatologie à Lomé (Togo)

**DOI:** 10.48327/mtsi.v4i3.2024.557

**Published:** 2024-08-05

**Authors:** Julienne Noude TECLESSOU, Yaovi Sénam EGOH, Koussake KOMBATE, Bayaki SAKA, Séfako AKAKPO, Palokinam PITCHE

**Affiliations:** 1 Service de dermatologie, Centre hospitalier universitaire (CHU) - Campus. Faculté des sciences de la santé, Université de Lomé, Togo; 2 Service de dermatologie, Centre hospitalier universitaire (CHU) - Sylvanus Olympio. Faculté des sciences de la santé, Université de Lomé, Togo

**Keywords:** Gale, Coût, Itinéraire thérapeutique, Qualité de vie, Automédication, Tradithérapeute, Pharmacien, Vendeur de rue, Infirmier, Généraliste, Dermatologue, CHU Campus, CHU Sylvanus Olympio, Centre de Gbossimé, Lomé, Togo, Afrique subsaharienne, Scabies, Cost, Therapeutic itinerary, Quality of life, Self-medication, Traditional healer, Pharmacist, Street vendor, Nurse, General practitioner, Dermatologist, CHU Campus, CHU Sylvanus Olympio, Gbossimé centre, Lomé, Togo, Sub-Saharan Africa

## Abstract

**Introduction:**

La gale est une parasitose cutanée souvent négligée qui peut altérer la qualité de vie des patients et dont la prise en charge peut être retardée dans certains pays pauvres d'Afrique par manque de personnels de santé formés ou par manque de moyens financiers des populations. Le but de cette étude était de décrire l'itinéraire thérapeutique des patients vus en consultation dermatologique à Lomé pour la gale, les coûts directs de la prise en charge et l'impact de cette affection sur leur qualité de vie.

**Patients et méthode:**

Il s'est agi d'une étude descriptive portant sur les patients chez qui le diagnostic de gale a été posé dans les services de dermatologie publics de Lomé du 1^er^ janvier au 31 décembre 2021. La qualité de vie a été évaluée avec le *Dermatology Life Quality Index* (DLQI).

**Résultats:**

Au total, 157 patients ont été inclus, dont 114 cas de gale clinique. L’âge médian des patients était de 26 ans et le sex-ratio (H/F) de 1,03. Parmi eux, 139 patients avaient un ou plusieurs parcours thérapeutiques avant la consultation dermatologique dont des automédications (80,9 %); des consultations chez des personnels soignants non dermatologues (62,4 %). Le coût des médicaments prescrits en consultation dermatologique pour le patient et son entourage était en moyenne de 19 817 FCFA (30,2 €). Le score DLQI moyen chez les adultes était de 7,4 et 34,9 % avaient une altération modérée de la qualité de vie. Le score moyen DQLI chez les enfants était de 7,2 et 71,4 % avaient une altération modérée de la qualité de vie.

**Conclusion:**

La gale est une parasitose qui altère la qualité de vie des patients. En absence de subvention et de moyens financiers pour les patients, sa prise en charge reste limitée ce qui pourrait contribuer à sa propagation.

## Abstract (en)

**Introduction:**

Scabies is an often-neglected skin parasitosis which can affect patients’ quality of life, and its treatment may be delayed in some poor African countries due to a lack of trained healthcare personnel or a lack of financial resources. The aim of this study was to describe the treatment-seeking paths of patients seen in dermatological consultations in Lomé for scabies, the direct costs of treatment and the impact of this condition on the quality of life of patients suffering from it.

**Patients and method:**

This was a descriptive and analytical study of patients diagnosed with scabies in one of Lomé’s dermatology departments from January 1 to December 31, 2021. The variables studied were sociodemographic; the patient's therapeutic course prior to the dermatology consultation; the direct cost of the present consultation and the patient's quality of life. The Dermatology Life Quality Index (DLQI) was used. Statistical analysis was performed with EPI Info software version 7.2.5.0.

**Results:**

A total of 157 patients were selected, including 114 cases of clinical scabies. The median age of patients was 26 years, and 29.3% were under 15 years of age. The sex-ratio M/F = 1.03. Out of them, 139 patients had one or more courses of treatment prior to the dermatological consultation, including self-medication (80.9%), consultations with non-dermatological healthcare personnel (62.4%). The average cost to the patient and family of drugs prescribed during the dermatological consultation was 19,817 CFA francs (30.2 €). The mean DQLI score for adults was 7.4, and 34.9% had moderate impairment of quality of life. The mean CDQLI score for children was 7.2, and 71.4 % had moderate impairment of quality of life.

**Conclusion:**

Scabies is a parasitosis that impair patients’ life quality. In the absence of subsidized treatment and financial resources for patients, treatment remains limited, which could contribute to the spread of the disease.

## Introduction

La gale est une affection dont les épidémies restent des fardeaux sanitaires et économiques considérables [1, 12]. Malgré les stratégies de lutte, elle constitue une préoccupation majeure dans les pays en voie de développement compte tenu de la surpopulation dans les logements qui augmente le risque de transmission, mais aussi du fait de l'accès limité aux soins de santé efficaces [2, 14]. Plus de 200 millions de personnes seraient atteints de gale dans le monde [7] et l'affection a été rapportée à des prévalences allant de 1,2 % à 5,88 % en milieu hospitalier [5, 8]. Des prévalences plus élevées ont été rapportées cependant en milieu scolaire ou communautaire allant de 13,33 à 17,8 % [9, 13]. Au Togo, la gale représentait 10,3 % des dermatoses en milieu carcéral en 2010 et constituait la première parasitose cutanée en milieu hospitalier (68,5 %) [15].

La prise en charge peut être retardée dans certains pays pauvres d'Afrique par manque de personnels de santé compétents ou par manque de moyens financiers des populations. Par ailleurs, le caractère contagieux de l'affection oblige le praticien à prendre en charge plusieurs membres de la famille, ce qui augmente également le coût du traitement pour les familles et pourrait limiter l'efficacité de cette prise en charge avec comme conséquence la persistance de petites épidémies communautaires. La persistance du prurit en absence de prise en charge rapide et efficace peut secondairement altérer la qualité de vie des patients qui en souffrent.

Le but de cette étude était de décrire l'itinéraire thérapeutique des patients vus en consultation dermatologique à Lomé pour la gale, les coûts directs de sa prise en charge et son impact sur la qualité de vie des patients qui en souffrent.

## Patients et méthode

Il s'est agi d'une étude descriptive portant sur les patients vus en consultation dans les services de dermatologie des centres hospitaliers universitaires (CHU) Campus et Sylvanus Olympio et le centre de dermatologie de Gbossimé à Lomé pendant une période de 12 mois (du 1^er^ janvier au 31 décembre 2021). Les CHU Campus et Sylvanus Olympio sont des hôpitaux de niveau 3 de la pyramide sanitaire à trois niveaux du Togo. Les services de dermatologie de ces CHU sont des unités de prestations de soins, de formations et de recherche scientifique en dermatologie.

Le centre de dermatologie de Gbossimé est un centre de prestations de soins en santé publique et communautaire qui abrite dans ses locaux un service de dermatologie s'occupant uniquement des consultations dermatologiques, le programme national de lutte contre la lèpre et une association allemande d'aide aux lépreux et tuberculeux : la DAHW *(Deutsche Lepra-und Tuberkulosehilfe e.V).* Les activités du service de dermatologie de Gbossimé sont assurées par le personnel du service de dermatologie du CHU Campus. A été inclus dans l’étude tout patient chez qui le diagnostic de la gale a été posé par un dermatologue de l'un des centres. Le diagnostic de gale était basé sur les critères diagnostiques de l'IACS (International Alliance for the Control of Scabies) [3].

Nous avons collecté des données :

- sociodémographiques : âge, sexe;
- sur le parcours thérapeutique des patients avant la consultation dermatologique : consultations médicales non spécialisées (coût des consultations et des médicaments achetés par le patient et son entourage éventuel); coût des visites chez des tradithérapeutes et autres donneurs de soins; coût de médicaments achetés tant en pharmacie que dans la rue (vendeur ambulant de médicaments) par propre initiative ou sous conseils (d'un pharmacien, vendeur en officine, ami, autre);
- sur les coûts directs de la présente consultation : coût de la consultation dermatologique et des médicaments prescrits;
- sur le revenu approximatif du patient (ou responsable de la famille);
- sur la qualité de vie des patients.

Le questionnaire DLQI *(Dermatology Life Quality Index)* adulte et le *Child DLQI* (CDLQI) ont été utilisés pour l’évaluation de la qualité de vie [4]. Le remplissage des questionnaires sur la qualité de vie s'est fait avec l'aide de traducteurs en langue locale pour les patients non lettrés ou ayant des difficultés à lire le français. Seuls les enfants âgés de 5 ans et plus et les adultes ont fait l'objet d’évaluation de la qualité de vie, le questionnaire CDLQI n’étant pas adapté pour les enfants de moins de cinq ans.

Nous n'avons pas inclus dans l'analyse de la qualité de vie les patients présentant une pathologie chronique (dermatologique ou non) pouvant aussi altérer leur qualité de vie. Nous avons obtenu le consentement écrit éclairé des participants. L'analyse statistique a été faite avec le logiciel EPI Info version 7.2.5.

Nous avons obtenu une autorisation de la direction du CHU Campus (MSPS/DRE/CUHCampus/0032/2020), du CHU Sylvanus Olympio (MSPS/DRE/CHU-SO/0011/2020) et de la responsable du service de dermatologie du centre de dermatologie de Gbossimé.

## Résultats

Au total, 157 des 1 619 patients vus dans les services de dermatologie présentaient des cas de gale, soit une prévalence globale de 9,7 %. Parmi ces 157 patients, 88 ont été recrutés dans le centre de dermatologie de Gbossimé (Tableau I). Leur âge médian était de 26 ans avec des extrêmes de 1 mois et 62 ans et 27 enfants étaient âgés de 5 à 15 ans (Tableau I). Le sex-ratio était de 1,03. Les pathologies chroniques retrouvées étaient principalement l'asthme (cinq patients) et l'hypertension artérielle (quatre patients) (Tableau I). Trente-deux (20,4 %) patients avaient une assurance maladie. Le nombre de sujets contacts des patients à traiter variait d'un à neuf avec une moyenne de 4,24. Le revenu mensuel moyen des patients ou du membre de la famille ayant en charge les frais des soins médicaux était estimé à 58 944 FCFA avec des extrêmes de 9 000 à 300 000 FCFA (89 €; extrêmes de 13 à 450 €). Vingt-et-un (13,3 %) avaient un revenu qui était égal à 3 fois le salaire minimum interprofessionnel garanti au Togo (SMIG) qui était de 35 000 FCFA (environ 53 €) au moment de l'enquête (Tableau I).

**Tableau I:** Aspects épidémiologiques, itinéraire thérapeutique et revenu mensuel des patients

	N	%
**Nombre de patients vus par centre de consultation n = 157**		
CHU Campus	33	21
CHU Sylvanus Olympio	36	22,9
Centre de dermatologie de Gbossimé	88	56,1
**Tranches d’âge (ans) n = 157**		
[0-5[ans	19	12,1
[5-15[ans	27	17,2
supérieur à 15	111	70,7
**Sexe**		
M	80	51
F	77	49
**Pathologie chronique n = 157**		
aucune	140	89,2
asthme	5	3,2
hypertension artérielle	4	2,5
épigastralgie/ulcère gastroduodénal	3	1,9
diabète	2	1,3
infection par le VIH	2	1,3
drépanocytose	1	0,6
**Itinéraire thérapeutique n = 139**		
**Consultation antérieure n=98**		
une consultation	42	42,9
deux consultations	29	29,6
trois consultations et plus	27	27,6
**Automédication n = 127**		
utilisation de baume/lait/crème	94	74
plantes médicinales	66	60
médicaments sur conseil en pharmacie	48	37,8
médicaments de rue*	38	29,9
**Revenu mensuel du patient ou du membre de famille en charge des soins médicaux**		
inférieur au SMIG	33	21,02
une fois le SMIG	41	26,11
deux fois le SMIG	27	17,2
trois fois le SMIG et plus	21	13,38
non précisé	35	22,3

* Médicament ayant un effet antihistaminique (cétirizine), anti-inflammatoire (ibuprofène), antiparasitaire (albendazol), antibiotique (amoxicilline)

### Itinéraire thérapeutique des patients avant la consultation dermatologique

La gale est communément appelée « *akpacus* » en langue locale éwé au sud du pays, « akpa » faisant allusion à l’écorce d'un arbre ou à un pneu de voiture. La personne souffrant de la gale se gratte au point que sa peau est comparable à de l’écorce ou à un pneu. En langue kotokoli au nord du pays, la gale est appelée « *kroussa-kroussa* », en rapport avec le prurit intense qu'induit la maladie. Il s'agit d'une maladie souvent considérée comme survenant chez des personnes à hygiène défectueuse, « *les gens sales* ».

Seuls 18 patients avaient consulté en dermatologie sans aucun parcours thérapeutique antérieur. Les 139 autres patients avaient une ou plusieurs étapes thérapeutiques. Parmi les 139 patients, 98 (70,5 %) avaient déjà consulté au moins une fois un personnel soignant (médecin généraliste, infirmier) avant la consultation dermatologique (Tableau I). Le coût moyen de ces consultations et les traitements prescrits par ces agents de santé était de 18 075 FCFA avec des extrêmes de 5 000 et 50 000 FCFA (coût moyen 27 €, extrêmes de 7 à 75 €).

Une automédication était adoptée par 127 des 139 (91,4 %) patients ayant des parcours thérapeutiques antérieurs (Tableau I). Le coût de l'automédication avant la consultation dermatologique était en moyenne de 5 972 FCFA (environ 9 €) avec des extrêmes de 100 à 25 000 FCFA (de 0,15 à 38 €). L'automédication était basée sur l'utilisation de baume mentholé ou autre crème/lait vendus sur le marché (74 %) dans le but de calmer le prurit, ou sur l'utilisation de décoctions de plantes de nature non précisée et autres thérapeutiques traditionnelles (60 %) (Tableau I).

### Coûts directs de la prise en charge de la gale

Le coût de la consultation dermatologique dans les centres inclus était de 3 000 FCFA (environs 4,5 €). Pour les patients ayant une assurance maladie, ce coût était de 600 FCFA soit environ 1 €. L'ivermectine n'est pas toujours disponible en officine au Togo. En cas de disponibilité, la boite de quatre comprimés est vendue environ 14 € et le flacon de 60 ml de benzoate de benzyle environ 4 €. Le traitement prescrit aux patients par les dermatologues était essentiellement le benzoate de benzyle (n = 132, soit 84,1 %), la crème de crotamiton (n = 25, 15,9 %) et la sumithrine (pyréthrinoïde) (100 %) pour la désinfection des linges. Ce traitement était prescrit au patient et aux sujets contacts. Seul le benzoate de benzyle est pris en charge par l'assurance maladie à un taux de remboursement de 80 %.

Le coût des médicaments prescrits en consultation dermatologique pour le patient et son entourage était en moyenne de 19 817 FCFA (30,2 €) avec des extrêmes de 13 000 à 52 430 FCFA (19,8 à 79,9 €). Quarante-sept (29,9 %) patients n'avaient pas honoré la totalité de l'ordonnance prescrite et 38 (24,3 %) avaient choisi de faire le traitement seuls sans les sujets contacts. En outre, 86 % des patients n'ayant pas honoré la totalité de l'ordonnance avaient un revenu mensuel inférieur ou égal à une fois le SMIG (p<0,0001).

### Qualité de vie

Seuls 106 patients de plus de 15 ans étaient exempts de toute pathologie chronique et 21 enfants âgés de 5 ans et plus sans antécédent de pathologie chronique ont été inclus.

Le score DQLI moyen chez les adultes était de 7,4 ± 3,19 et 34,9 % avaient une altération modérée de la qualité de vie (Tableau II). La gale avait impacté le sommeil « énormément » et « beaucoup » chez respectivement 14,3 % et 57,1 % des 106 patients de plus de 15 ans. Au cours des 7 derniers jours, l’état de la peau avait été « beaucoup » la cause d'un certain embarras ou d'un manque d'assurance chez 25,5 % des patients de plus de 15 ans (Tableau III).

**Tableau II:** Évaluation de la qualité de vie chez l'enfant et chez l'adulte selon le score DLQI (n = 106)

Score DLQI	N (%)	Effet sur la qualité de vie
**Enfants 5 à 15 ans**		
0-1	0	aucun
2-6	6 (28,6)	faible
7-12	15 (71,4)	modéré
13-18	0	très important
19-30	0	extrêmement important
Adultes > 15 ans		
0-1	3 (2,8)	aucun
2-5	43 (40,6)	faible
6-10	37 (34,9)	modéré
11-20	21 (19,8)	important
21-30	2 (1,9)	très important

**Tableau III:** Évaluation de la qualité de vie chez l'adulte selon le score DLQI (n = 85)

Questionnaire DLQI adulte	Énormément n (%)	Beaucoup n (%)	Un peu n (%)	Pas du tout n (%)	Non concerné (e) n (%)
Au cours des 7 derniers jours, votre peau vous a-t-elle démangé ou piqué, ou bien avez-vous ressenti des irritations ou de la douleur?	44 (41,5)	50 (47,2)	12 (11,3)	0	0
Au cours des 7 derniers jours, l’état de votre peau a-t-il été la cause d'un certain embarras ou d'un manque d'assurance?	10 (9,4)	27 (25,5)	38 (35,8)	21 (19,8)	10 (9,4)
Au cours des 7 derniers jours, l’état de votre peau vous a-t-il gêné pour faire vos courses ou vous occuper de votre domicile ou de votre jardin?	3(2,8)	20 (18,9)	23 (21,7)	43 (40,6)	17 (16,03)
Au cours des 7 derniers jours, l’état de votre peau a-t-il influencé le choix de vos vêtements?	6 (5,7)	16 (15,1)	25 (23,6)	52 (49,1)	7(6,6)
Au cours des 7 derniers jours, l’état de votre peau a-t-il eu un impact sur vos activités sociales ou de loisirs?	6 (5,7)	15 (14,2)	26 (24,5)	54 (50,9)	5 (4,7)
Au cours des 7 derniers jours, l’état de votre peau a-t-il rendu difficile la pratique d'un sport?	3 (5,3)	4 (7)	8 (7,5)	39 (36,8)	52 (49)
Au cours des 7 derniers jours, l’état de votre peau vous a-t-il empêché de travailler ou d’étudier?	Oui				
Si vous avez répondu par « Non » : au cours des 7 derniers jours, l’état de votre peau a-t-il été un problème au travail ou dans vos études?	11 (10,4)	11 (10,4)	7 (6,9)	46 (43,4)	31 (29,2)
Au cours des 7 derniers jours, l’état de votre peau a-t-il été à l'origine de difficultés avec votre compagnon (compagne), vos amis proches ou les membres de votre famille?	4 (3,8)	8 (7,5)	12 (11,3)	82 (77,3)	0
Au cours des 7 derniers jours, l’état de votre peau a-t-il engendré des difficultés d'ordre sexuel?	4 (3,8)	8 (7,5)	8 (7,5)	45(42,5	41 (38,7)
Au cours des 7 derniers jours, votre traitement pour la peau a-t-il causé des problèmes? Par exemple, a-t-il eu un impact sur l'ordre ou la propreté de la maison? Ou bien, le traitement prend-il trop de temps?	1 (1,8)	3(2,8)	8 (7,5)	46 (43,4)	48 (45,3)

Le score moyen CDQLI chez les enfants était de 7,2 ± 2,79 et 71,4 % avaient une altération modérée de la qualité de vie. Au cours de la dernière semaine, la peau avait démangé, ou fait mal « énormément » à 38,1 % des 21 enfants et « beaucoup » à 47,6 % des 21 enfants. Aussi, 57 % de ces enfants disaient être « beaucoup » gênés ou mal à l'aise, malheureux ou tristes à cause de la gale; 9,5 % avaient « beaucoup » de conséquences sur le travail à l’école et 14,3 % avaient « énormément » mal dormi à cause de leurs problèmes de peau (Tableau IV).

**Tableau IV:** Évaluation de la qualité de vie chez l'enfant selon le score DLQI (n = 21)

Questionnaire DLQI enfant	Énormément n (%)	Beaucoup n (%)	Un peu n (%)	Pas du tout n (%)
Au cours de la semaine dernière, est-ce que ta peau t'a démangé ou gratté, ou t'a fait mal?	8 (38,1)	10 (47,6)	3 (14,3)	0
Au cours de la semaine dernière, est-ce que tu as été gêné ou mal à l'aise, malheureux ou triste à cause de tes problèmes de peau?	0	12 (57,1)	6 (28,6)	3 (14,3)
Au cours de la semaine dernière, est-ce que tes problèmes de peau ont changé tes relations avec tes copains?	0	0	6 (28,6)	15 (71,4)
Au cours de la semaine dernière, est-ce que tu as dû te changer ou porter des chaussures ou des vêtements différents ou spéciaux à cause de tes problèmes de peau?	0	2 (9,5)	3 (14,3)	16 (76,2)
Au cours de la semaine dernière, est-ce que tes problèmes de peau t'ont gêné pour sortir jouer, ou faire les choses qui t'intéressent?	0	0	5 (23,8)	16 (76,2)
Au cours de la semaine dernière, est-ce que tu as évité d'aller nager ou de faire du sport à cause de tes problèmes de peau?	0	0	0	21 (100)
Si tu avais école : au cours de la semaine dernière, est-ce que tes problèmes de peau ont eu des conséquences sur ton travail à l’école?	0	2 (9,5)	5 (23,8)	14 (66,7)
Si tu étais en vacances : au cours de la semaine dernière, est-ce que tes problèmes de peau t'ont empêché de passer de bonnes vacances?	0	4 (19)	6 (28,6)	11 (52,4)
Au cours de la semaine dernière, est-ce qu’à cause de tes problèmes de peau tu as été embêté (e) par les autres?	1 (4,8)	7 (33,3)	8 (38,1)	5 (23,8)
Au cours de la semaine dernière, est-ce que tu as mal dormi à cause de tes problèmes de peau?	3 (14,3)	12 (57, 1)	5 (23,8)	1 (4,8)
Au cours de la semaine dernière, est-ce que le traitement pour ta peau t'a posé des problèmes?	0 (0,0)	0 (0,0)	1 (4,8)	20 (95,2)

## Discussion

Cette étude nous a permis de décrire l'itinéraire thérapeutique des patients souffrant de gale avant une consultation dermatologique, itinéraire qui était constitué de rencontres avec des soignants non dermatologues, d'automédications et de visites chez des tradithérapeutes. Le coût direct de la prise en charge de la gale par le dermatologue a été évalué à environ 30 €. La gale altérait la qualité de vie des patients qui en souffraient, avec principalement un effet sur leur sommeil. Notre étude comporte des limites et des biais.

Nous n'avons pas d’évaluation du coût indirect de la gale qui pourrait aussi expliquer le fait que les patients n'honorent pas la totalité des prescriptions dermatologiques.

Nous n'avons pas recherché la composition des traitements utilisés par les patients lors des phytothérapies ou chez les tradithérapeutes.

Enfin, les raisons pour lesquelles les patients avaient eu plusieurs recours thérapeutiques n'ont pas été recherchées.

La majorité (88/157) des patients avait consulté dans le service de dermatologie de Gbossimé, bien que le coût de la consultation soit identique dans les trois centres. Ceci peut être dû à l'accessibilité géographique de ce centre, qui est un centre communautaire situé dans un quartier populaire au cœur de la ville de Lomé. Par ailleurs, l'idée que le centre de dermatologie soit une référence en matière de prise en charge des dermatoses dans la population n'est pas à exclure.

Seuls 20,4 % des patients avaient une assurance maladie. Il est difficile d’évaluer la proportion de personnes ayant une assurance maladie au Togo compte tenu de l'existence de plusieurs régimes d'assurance selon le secteur d'exercice, les employeurs du secteur privé n'ayant pas l'obligation de contracter une assurance maladie à leurs employés. On estime à environ 500 000 le nombre de personnes bénéficiant d'une assurance maladie en 2022, essentiellement des fonctionnaires. Plus de la moitié (62,4 %) avait consulté au moins une fois un agent de santé avant la consultation dermatologique et 80,9 % avaient eu recours à une automédication. Une précédente étude sur les connaissances, attitudes et pratiques des personnels soignants non dermatologues face à la gale à Lomé, révélait des attitudes et des pratiques insuffisantes : 54,6 % des agents de santé prescrivaient essentiellement des antihistaminiques pour la prise en charge de la gale et 69,9 % ne prescrivaient pas de traitement pour les sujets contacts [15]. Parmi les patients étudiés, 29,9 % n'avaient pas honoré la totalité de l'ordonnance prescrite par le dermatologue. L'une des raisons explicatives pourrait être liée aux dépenses antérieures à la consultation dermatologique.

La gale constitue une parasitose cutanée souvent négligée ou méconnue, dont le coût direct ou indirect sur la population devra être évalué afin de définir de meilleures stratégies de prise en charge. L’évaluation de ces coûts permettra qu'elle ne soit plus aussi négligée à l'avenir qu'elle ne l'est aujourd'hui [10]. Le coût des médicaments prescrits par le dermatologue au patient était en moyenne de 30 €.

Le coût moyen des consultations et des médicaments prescrits par des agents de santé au patient seul avant la consultation dermatologique (27 € en moyenne) était proche du coût des médicaments prescrits par le dermatologue au patient et au sujet contact (30,2 € en moyenne). La gale étant une dermatose courante pouvant être traitée par des médecins généralistes, une bonne connaissance de la prise en charge de cette affection par les personnels soignants non dermatologues devrait permettre la guérison des patients, limiter la propagation de l'affection et éviter l'errance des patients. L'automédication, dont le coût (9 € en moyenne) était trois fois inférieur au coût de la prise en charge par les dermatologues (30,2 €), est totalement inefficace, ce qui explique le recours au dermatologue après ce parcours. Ces automédications étaient basées sur les recours à des plantes médicinales, des médicaments de rue de provenance et de compositions souvent douteuses, et pharmacologiquement inefficaces et inadéquates. Bien que nous n'ayons pas recherché la composition exacte des traitements utilisés par les patients lors des phytothérapies ou chez les tradithérapeutes, nous craignons que ces thérapeutiques aient des conséquences à court, moyen ou long terme sur la santé des patients notamment en matière de toxicité (hépatique, rénale, et autres). Le score DLQI et CDLQI était respectivement de 7,4 et 7,2 et 34,9 % des adultes avaient une altération modérée de la qualité de vie. Nos résultats sont proches de ceux de Ly *et al.* qui avaient trouvé au Sénégal un DLQI et CDLQI moyen de 7,8 et 8,1 [11). Jin-gang *et al.* avaient pour leur part relevé un score moyen de 10,09 avec une altération modérée de la qualité de vie chez 37,50 % des patients [6]. Dans une étude au Brésil, Worth *et al.* utilisant un questionnaire modifié et adapté du DQLI, avaient constaté un score moyen de 3 chez l'adulte et 2 chez l'enfant [16]. La gale affecte donc d'une façon générale la qualité de vie des patients.

## Conclusion

La gale est due à un ectoparasite dont les manifestations cliniques altèrent la qualité de vie des patients et suscitent plusieurs recours thérapeutiques avant la consultation dermatologique. A notre connaissance, aucune étude au Togo ou dans la sous-région n'a évalué le coût direct ou indirect de la gale. Notre étude montre qu'elle est responsable d'une altération modérée de la qualité de vie chez les patients. Une bonne prise en charge des patients et sujets contacts permettra de limiter la propagation de la maladie.

## Contribution des auteurs

Julienne Noude TECLESSOU : conception, collecte des données, rédaction du manuscrit Yaovi Sénam EGOH : collecte de données, rédaction du manuscrit

Koussake KOMBATE : rédaction du manuscrit Séfako AKAKPO : rédaction du manuscrit Bayaki SAKA : rédaction du manuscrit Palokinam PITCHE : relecture

## Déclaration d'intérêt

Les auteurs ne rapportent aucun conflit d'intérêt.
