# Supplementary material for: Therapeutic itinerary, direct costs, and life quality of patients with human scabies followed in dermatology in Lomé (Togo)
Source: Med Trop Sante Int. 2024 Aug 5;4(3):mtsi.v4i3.2024.557. [Article in French] doi: 10.48327/mtsi.v4i3.2024.557 (PMC11809070; doi:10.48327/mtsi.v4i3.2024.557)
Supplement: Related texts

# Research article: Therapeutic itinerary, direct costs, and life quality of patients with human scabies followed in dermatology in Lomé (Togo)

## Introduction

Scabies is a disease whose epidemics remain a considerable health and economic burden [1, 12]. Despite control strategies, it remains a major concern in developing countries, due to overcrowding in housing, which increases the risk of transmission, and limited access to effective healthcare [2, 14]. More than 200 million people are thought to suffer from scabies worldwide [7], and the condition has been reported at prevalences ranging from 1.2% to 5.88% in hospital settings [5, 8]. However, higher prevalences have been reported in school and community settings, ranging from 13.33% to 17.8% [9, 13]. In Togo, scabies accounted for 10.3% of dermatoses in jails in 2010, and was the leading cutaneous parasitosis in hospitals (68.5%) [15].

In some poor African countries, treatment may be delayed due to a lack of trained health personnel or financial resources. In addition, the contagious nature of the condition obliges the practitioner to treat several members of the family, which also increases the cost of treatment for families and could limit the effectiveness of this treatment, resulting in the persistence of small community epidemics. The persistence of pruritus in the absence of prompt and effective management may secondarily impair the quality of life of patients suffering from it.

The aim of this study was to describe the therapeutic itinerary of patients seen in dermatological consultations in Lomé for scabies, the direct costs of its management and its impact on the quality of life of patients suffering from it.

## Patients and method

This was a descriptive study of patients seen in consultation in the dermatology departments of the Campus and Sylvanus Olympio university hospitals (CHU) and the Gbossimé dermatology center in Lomé over a 12-month period (January 1 to December 31, 2021). CHU Campus and CHU Sylvanus Olympio are level 3 hospitals in Togo's three-tiered health pyramid. Their dermatology departments provide care, training and scientific research in dermatology. The Gbossimé dermatology center is a public health and community care center, which houses a dermatology department dedicated exclusively to dermatological consultations, the national leprosy control program and a German association for leprosy and tuberculosis sufferers: DAHW *(Deutsche Lepra-und Tuberkulosehilfe e.V).* The Gbossimé dermatology department is staffed by personnel from the CHU Campus dermatology department. All patients diagnosed with scabies by a dermatologist at one of the centers were included in the study. The diagnosis of scabies was based on IACS (International Alliance for the Control of Scabies) diagnostic criteria [3].

We collected data on:

- sociodemographic features: age, sex;
- patient's treatment-seeking path prior to the dermatological consultation: non-specialized medical consultations (cost of consultations and drugs purchased by the patient and any family and friends); cost of visits to traditional healers and other caregivers; cost of drugs purchased both in pharmacies and on the street (ambulant drug salesman) on the patient's own initiative or under advice (from a pharmacist, pharmacy salesman, friend, other);
- direct costs of the present consultation: cost of the dermatological consultation and prescribed medication;
- patient's (or family member's) approximate income;
- patient's quality of life.

The Dermatology Life Quality Index (DLQI) adult questionnaire and the Children's DLQI (CDLQI) were used to assess quality of life [4]. The quality-of-life questionnaires were completed with the help of local-language translators for patients who were illiterate or had difficulty reading French. Only children aged 5 and over and adults were assessed for quality of life, as the CDLQI questionnaire is not suitable for children under 5. We did not include patients with a chronic pathology (dermatological or not) that could also alter their quality of life in the quality-of-life analysis. Written informed consent was obtained from participants. Statistical analysis was performed using EPI Info software version 7.2.5.

We obtained authorization from the management of CHU Campus (MSPS/DRE/CUHCampus/0032/2020), CHU Sylvanus Olympio (MSPS/DRE/CHU-SO/0011/2020) and the head of the dermatology department of the Gbossimé dermatology center.

## Results

A total of 157 of the 1,619 patients seen in the dermatology departments had cases of scabies, representing an overall prevalence of 9.7%. Of these 157 patients, 88 were recruited from the Gbossimé dermatology center (Table I). Their median age was 26 years, with extremes of 1 month and 62 years, and 27 children were aged between 5 and 15 years [FM1] (Table I). The sex ratio was 1.03. Chronic pathologies were mainly asthma (5 patients) and arterial hypertension (4 patients) (Table I). Thirty-two (20.4%) patients had health insurance.

**Table I:** Epidemiological aspects, therapeutic itinerary and patients’ monthly incom

	N	%
**Nombre de patients vus par centre de consultation n = 157**		
CHU Campus	33	21
CHU Sylvanus Olympio	36	22,9
Centre de dermatologie de Gbossimé	88	56,1
**Tranches d’âge (ans) n = 157**		
[0-5[ans	19	12,1
[5-15[ans	27	17,2
supérieur à 15	111	70,7
**Sexe**		
M	80	51
F	77	49
**Pathologie chronique n = 157**		
aucune	140	89,2
asthme	5	3,2
hypertension artérielle	4	2,5
épigastralgie/ulcère gastroduodénal	3	1,9
diabète	2	1,3
infection par le VIH	2	1,3
drépanocytose	1	0,6
**Itinéraire thérapeutique n = 139**		
**Consultation antérieure n=98**		
une consultation	42	42,9
deux consultations	29	29,6
trois consultations et plus	27	27,6
**Automédication n = 127**		
utilisation de baume/lait/crème	94	74
plantes médicinales	66	60
médicaments sur conseil en pharmacie	48	37,8
street medications*	38	29,9
**Revenu mensuel du patient ou du membre de famille en charge des soins médicaux**		
inférieur au SMIG	33	21,02
une fois le SMIG	41	26,11
deux fois le SMIG	27	17,2
trois fois le SMIG et plus	21	13,38
non précisé	35	22,3

* Antihistamine (cetirizine), anti-inflammatory (ibuprofen), antiparasitic (albendazol), antibiotic (amoxicillin).

The number of patient contacts ranged from 1 to 9, with an average of 4.24. The average monthly income of patients or family members paying for medical care was estimated at 58,944 FCFA with extremes from 9,000 to 300,000 FCFA (89€; extremes from 13 to 450€). Twenty-one (13.3%) had an income equal to 3 times the guaranteed minimum wage in Togo (SMIG), which was 35,000 FCFA (around 53€) at the time of the survey (Table I).

### Patients’ therapeutic itinerary prior to dermatological consultation

Scabies is commonly called “akpacus” in the local Ewe language in the south of the country, “akpa” referring to the bark of a tree or a car tire. A person suffering from scabies scratches to the point where their skin is comparable to bark or a tire. In the northern Kotokoli language, scabies is called “kroussa-kroussa”, in reference to the intense pruritus induced by the disease. It is a disease often considered to occur in people with poor hygiene, “dirty people”.

Only 18 patients had consulted a dermatologist with no previous history of treatment. The remaining 139 patients had undergone one or more therapeutic steps. Of the 139 patients, 98 (70.5%) had already consulted a healthcare personnel (GP, nurse) at least once prior to the dermatological consultation (Table I). The average cost of these consultations and the treatments prescribed by these health workers was 18,075 FCFA, with extremes of 5,000 and 50,000 FCFA (average cost 27€, extremes 7 to 75€).

Self-medication was adopted by 127 of the 139 (91.4%) patients with previous treatment histories (Table I). The cost of self-medication prior to dermatological consultation averaged 5,972 FCFA (approx. 9€), with extremes ranging from 100 to 25,000 FCFA (0.15 to 38€). Self-medication was based on the use of menthol balm or other skin cream/milk sold on the market (74%) to soothe pruritus, or on the use of unspecified plant decoctions and other traditional therapies (60%) (Table I).

### Direct costs of scabies management

The cost of a dermatological consultation in the included centers was 3,000 FCFA (around 4.5€). For patients with health insurance, this cost was 600 FCFA (around 1€). Ivermectin is not always available in pharmacies in Togo. When it is available, a box of four tablets is sold for around 14€, and a 60 ml bottle of benzyl benzoate for around 4€. The treatment prescribed to patients by dermatologists was mainly benzyl benzoate (n = 132, 84.1%), crotamiton cream (n = 25, 15.9%) and sumithrin (pyrethroid) (100%) for disinfecting linen. This treatment was prescribed to the patient and contact subjects. Only benzyl benzoate is reimbursed by the health insurance at a rate of 80%.

The average cost to the patient and their family of the drugs prescribed during the dermatological consultation was 19,817 FCFA (30.2€), with extremes ranging from 13,000 to 52,430 FCFA (19.8€ to 79.9€). Forty-seven (29.9%) patients failed to pay the full prescription, and 38 (24.3%) chose to carry out the treatment on their own, without their contacts. In addition, 86% of patients who failed to pay the full prescription had a monthly income less than or equal to one time the minimum wage (p<0.0001).

### Quality of life

Only 106 patients over 15 years of age were free of any chronic pathology, and 21 children aged 5 and over with no history of chronic pathology were included.

The mean DQLI score for adults was 7.4 ± 3.19, and 34.9% had moderate impairment of quality of life (Table II). Scabies had impacted sleep “very much” and “a lot” in 14.3% and 57.1% of the 106 patients over 15 years of age respectively. In the last 7 days, skin condition had been the cause of “a lot” of embarrassment or insecurity in 25.5% of patients over 15 (Table III).

**Table II:** Assessment of quality of life in children and adults according to DLQI score (n = 106)

DLQI score	N (%)	Effect on quality of life
**Children aged 5 to 15**		
0-1	0	none
2-6	6 (28,6)	low
7-12	15 (71,4)	moderate
13-18	0	very high
19-30	0	extremely important
**Adults > 15 years**		
0-1	3 (2,8)	none
2-5	43 (40,6)	low
6-10	37 (34,9)	moderate
11-20	21 (19,8)	high
21-30	2 (1,9)	very high

**Table III:** Assessment of quality of life in adults according to DLQI score (n = 85)

Adult DLQI questionnaire	Very much n (%)	A lot n (%)	A little n (%)	Not at all (%) n (%)	Not relevant (%) n (%)
In the last 7 days, has your skin itched, stung, irritated or hurt?	44 (41,5)	50 (47,2)	12 (11,3)	0	0
In the last 7 days, has the condition of your skin made you feel embarrassed or insecure?	10 (9,4)	27 (25,5)	38 (35,8)	21 (19,8)	10 (9,4)
In the last 7 days, has your skin condition made it difficult for you to do your shopping, look after your home or garden?	3(2,8)	20 (18,9)	23 (21,7)	43 (40,6)	17 (16,03)
In the last 7 days, has your skin condition influenced your choice of clothes?	6 (5,7)	16 (15,1)	25 (23,6)	52 (49,1)	7(6,6)
In the last 7 days, has your skin condition affected your social or leisure activities?	6 (5,7)	15 (14,2)	26 (24,5)	54 (50,9)	5 (4,7)
In the last 7 days, has your skin condition made it difficult for you to practice a sport?	3 (5,3)	4 (7)	8 (7,5)	39 (36,8)	52 (49)
In the last 7 days, has your skin condition prevented you from working or studying?	No : 106 (100 %)				
If you answered “No”: in the last 7 days, has your skin condition been a problem at work or in your studies?	11 (10,4)	11 (10,4)	7 (6,9)	46 (43,4)	31 (29,2)
In the last 7 days, has your skin condition caused difficulties with your partner, close friends or family members?	4 (3,8)	8 (7,5)	12 (11,3)	82 (77,3)	0
In the last 7 days, has your skin condition caused sexual difficulties?	4 (3,8)	8 (7,5)	8 (7,5)	45(42,5	41 (38,7)
In the last 7 days, has your skin treatment caused any problems? For example, has it had an impact on the order or cleanliness of your home? Or is the treatment taking too long?	1 (1,8)	3(2,8)	8 (7,5)	46 (43,4)	48 (45,3)

The mean CDQLI score in children was 7.2 ± 2.79, and 71.4% had moderate impairment of quality of life. In the last week, the skin had itched or hurt “very much” in 38.1% of the 21 children, and “a lot” in 47.6% of the 21 children. Also, 57% of these children said they were “a lot” embarrassed or uncomfortable, unhappy or sad because of the scabies; 9.5% had “a lot” affected work at school and 14.3% had “very much” slept badly because of their skin problems (Table IV).

**Table IV:** Assessment of quality of life in children according to DLQI score (n = 21)

Child DLQI questionnaire	Very much n (%)	A lot n (%)	A little n (%)	Not at all (%) n (%)
Over the past week, has your skin itched, scratched or hurt?	8 (38,1)	10 (47,6)	3 (14,3)	0
In the past week, have you been embarrassed or uncomfortable, unhappy or sad because of your skin problems?	0	12 (57,1)	6 (28,6)	3 (14,3)
In the past week, have your skin problems changed your relationships with your friends?	0	0	6 (28,6)	15 (71,4)
In the past week, have you had to change or wear different or special clothes or shoes because of your skin problems?	0	2 (9,5)	3 (14,3)	16 (76,2)
In the past week, have your skin problems made it difficult for you to go out and play, or to do the things that interest you?	0	0	5 (23,8)	16 (76,2)
In the past week, have you avoided going swimming or playing sports because of your skin problems?	0	0	0	21 (100)
If you were at school: over the past week, have your skin problems affected your work at school?	0	2 (9,5)	5 (23,8)	14 (66,7)
If you were on vacation: in the last week, did your skin problems prevent you from having a good vacation?	0	4 (19)	6 (28,6)	11 (52,4)
In the past week, were you bothered by other people because of your skin problems?	1 (4,8)	7 (33,3)	8 (38,1)	5 (23,8)
In the last week, have you slept badly because of your skin problems?	3 (14,3)	12 (57, 1)	5 (23,8)	1 (4,8)
Over the past week, have you had any problems with your skin treatment?	0 (0,0)	0 (0,0)	1 (4,8)	20 (95,2)

## Discussion

This study enabled us to describe the therapeutic itinerary of patients suffering from scabies prior to a dermatological consultation, an itinerary that consisted of encounters with non-dermatological carers, self-medication and visits to traditional healers. The direct cost of scabies treatment by a dermatologist was estimated at around 30€. Scabies altered the quality of life of patients suffering from it, mainly affecting their sleep.

Our study has limitations and biases.

We have no evaluation of the indirect cost of scabies, which could also explain why patients do not honor all dermatological prescriptions. We did not investigate the composition of the treatments used by patients during herbal treatments or with traditional healers.

Lastly, we did not investigate the reasons why patients had used several treatments.

The majority (88/157) of patients had consulted the dermatology department at Gbossimé, although the cost of consultation was identical in all three centers. This may be due to the geographical accessibility of this center, which is a community center located in a working-class district in downtown Lomé. In addition, the idea that the dermatology center is a reference for the management of dermatoses in the population cannot be ruled out.

Only 20.4% of patients had health insurance. It is difficult to assess the proportion of people with health insurance in Togo, given the existence of different insurance schemes depending on the sector in which they work, and the fact that private-sector employers are not required to provide health insurance for their employees. It is estimated that around 500,000 people will be covered by health insurance in 2022, most of them civil servants.

More than half (62.4%) had consulted a health personnel at least once prior to the dermatological consultation, and 80.9% had used self-medication. A previous study on the knowledge, attitudes and practices of non-dermatological healthcare personnal in relation to scabies in Lomé revealed inadequate attitudes and practices: 54.6% of healthcare personnel mainly prescribed antihistamines for the management of scabies, and 69.9% did not prescribe any treatment for contact subjects [15]. Of the patients studied, 29.9% had not paid the dermatologist's prescription in full. One of the reasons for this could be related to expenses incurred prior to the dermatological consultation.

Scabies is a skin parasitosis that is often neglected or unrecognized, and its direct or indirect cost to the population needs to be assessed in order to define better management strategies. Assessing these costs will ensure that scabies is not as neglected in the future as it is today [10]. The cost of drugs prescribed by the dermatologist to the patient averaged 30€.

The average cost of consultations and drugs prescribed by health workers to the patient alone before the dermatological consultation (27€ on average) was close to the cost of drugs prescribed by the dermatologist to the patient and contact subject (30.2€ on average). As scabies is a common dermatosis that can be treated by general practitioners, a good knowledge of how to manage the condition among non-dermatologist healthcare personnel should help patients recover, limit the spread of the condition and avoid patients wandering off. Self-medication, the cost of which (9€ on average) was three times lower than the cost of treatment by dermatologists (30.2€), was totally ineffective, which explains why patients turned to dermatologists after seeking treatment previously. Self-medication was based on the use of medicinal plants, street medicines of dubious origin and composition, and pharmacologically ineffective and inadequate. Although we have not researched the exact composition of the treatments used by patients during herbal therapies or by traditional practitioners, we fear that these therapies may have short-, medium- or long-term consequences on patients’ health, particularly in terms of toxicity (hepatic, renal, etc.).

The DLQI and CDLQI scores were 7.4 and 7.2 respectively, and 34.9% of adults had moderate impairment of quality of life. Our results are close to those of Ly et al. who found in Senegal a mean DLQI and CDLQI of 7.8 and 8.1 [11]. Jin-gang *et al.* found a mean score of 10.09, with moderate impairment of quality of life in 37.50% of patients [6]. In a study in Brazil, Worth et al., using a modified questionnaire adapted from the DQLI, found a mean score of 3 in adults and 2 in children [16]. Scabies therefore generally affects patients’ quality of life.

## Conclusion

Scabies is caused by an ectoparasite, the clinical manifestations of which impair patients’ quality of life and give rise to several therapeutic remedies prior to dermatological consultation. To our knowledge, no study in Togo or in the sub-region has evaluated the direct or indirect cost of scabies. In our study, scabies was responsible for a moderate deterioration in patients’ quality of life. Proper management of patients and contacts will help limit the spread of the disease.

## Authors’ contributions

Julienne Noude TECLESSOU: study design, data collection, drafting of manuscript Yaovi Sénam EGOH: data collection, drafting of manuscript

Koussake KOMBATE: drafting of manuscript Séfako AKAKPO: drafting of manuscript Bayaki SAKA: drafting the manuscript Palokinam PITCHE: proofreading

## Conflict of interest

The authors declare no conflict of interests.
